# Correlation between Chemical Characterization and Biological Activity: An Urgent Need for Human Studies Using Extra Virgin Olive Oil

**DOI:** 10.3390/antiox11020258

**Published:** 2022-01-28

**Authors:** Stefania De Santis, Maria Lisa Clodoveo, Filomena Corbo

**Affiliations:** 1 Department of Pharmacy-Pharmaceutical Science, University of Bari Aldo Moro, 70126 Bari, Italy; filomena.corbo@uniba.it; 2 Department of Interdisciplinary Medicine, University of Bari Aldo Moro, 70124 Bari, Italy; marialisa.clodoveo@uniba.it

**Keywords:** extra virgin olive oil, polyphenols, chemical characterization, antioxidant activity, anti-inflammatory potential, human studies, health claim

## Abstract

Extra virgin olive oil (EVOO) is one of the most important functional foods from the Mediterranean Diet due to its beneficial effect on human health in terms of prevention and/or adjuvant treatment of different pathological conditions. The positive effects linked to EVOO consumption are not only due to its major (monounsaturated fatty acids), but also to its minor components (phenolics), whose roles were greatly re-evaluated in the last years. Notwithstanding the huge number of studies demonstrating the antioxidant, anti-inflammatory and anti-cancer properties of EVOO’s phenolic compounds, only their antioxidant ability was supported by a Health Claim. However, to bear the claim, a specific phenolic composition is needed, thus reinforcing the need to correlate the characterization of the phenolic compounds to their biological activity. In fact, although the chemical characterization of VOO’s phenolic compounds was extensively studied, its correlation with biological effects is only partially investigated; this is especially true for human studies. This review aims to study the correlation between the chemical characterization of EVOO’s phenolics and the biological effects in terms of antioxidant/anti-inflammatory potentials, with a focus on the human studies and the relative concern on getting a specific Health Claim.

## 1. Introduction

Extra virgin olive oil (EVOO) is one of the most important functional foods from the Mediterranean Diet (MD) and its consumption is linked to a reduction in the risk of cardiovascular and chronic inflammatory diseases [1,2,3,4]. The beneficial effect of olive oil on human health depends on the high content of both the monounsaturated fatty acids (MUFAs) (80% of its total lipid composition) and the phenolic compounds, polar molecules, whose extremely variable concentration is a function of agronomic and technological conditions [5,6,7,8]. Even if present at a low extent (1–2% of EVOO), the phenolic compounds were re-evaluated in recent last years, as indicated by the huge number of papers demonstrating their efficacy in the prevention and/or as adjuvant treatment for different pathological conditions thanks to the antioxidant, anti-inflammatory and anti-cancer properties [9,10,11]. Importantly, some of these papers were considered for the establishment of a specific Health Claim by the EFSA (European Food Safety Authority). Notwithstanding the multitude of studies underlying the anti-inflammatory and anti-cancer properties of the phenolic compounds, only the antioxidant ability is fully established for these compounds, even if a specific chemical composition is required (see below). This requirement reinforces the need to correlate the characterization of the phenolic compounds to their biological activity; this is especially true for human studies, even if this correlation is often missing in these cases. In fact, although the chemical characterization of VOO’s phenolic compounds was extensively studied with consolidated techniques [12,13], its correlation with the biological effects, mainly linked to the antioxidant and/or the anti-inflammatory properties, is only partially investigated.

The aim of this review is to examine the state-of-art of the human studies in which a detailed chemical characterization of polyphenols from EVOO/VOO (only administered in the raw form) was correlated to the biological effects in terms of antioxidant and anti-inflammatory potentials. 

## 2. Correlation among EVOO Polyphenols Chemical Characterization, Antioxidant Activity and Biological Effects in Human Studies

The antioxidant activity of VOO’s polyphenols was studied in different pathological contexts. One of the most investigated contexts of VOO’s biological activities in humans is cardiovascular disease (CVD) prevention, that relies on different mechanisms, such as the improvement of the lipid profile by increasing high density lipoprotein (HDL)-cholesterol and reducing low density lipoprotein (LDL)-cholesterol and triglycerides (TAG) [14], the reduction of oxidative stress, and the inhibition of human lipoprotein oxidation [15]. On this basis, EFSA approved a Health Claim on 2011 establishing a cause–effect relationship between the consumption of olive oil polyphenols (standardized by the content of hydroxytyrosol and its derivatives) and the protection of LDL particles from the oxidative damage. To bear this claim, OO has to contain 5 mg of hydroxytyrosol and its derivatives (e.g., oleuropein complex and tyrosol) per 20 g of OO [16]. This Health Claim was accepted by the European Union (EU) one year later [17]. One of the most well-structured studies taken into account by the EFSA Panel for this Health Claim is the EUROLIVE study, a randomized, cross-over, controlled intervention trial in which 200 healthy subjects were enrolled for a sustained consumption of VOO with high (366 mg/kg), moderate (164 mg/kg) and low (2.7 mg/kg) polyphenols content (HPC, MPC and LPC, respectively) [15]. The authors observed a dose-dependent effect between the increase of polyphenols content in VOO and the decrease in LDL peroxidation, conjugated dienes and hydroxy fatty acids, and an increase in HDL-cholesterol concentrations. As reported before, the maintenance of normal blood HDL-cholesterol concentrations is another beneficial effect evaluated by the EFSA Panel for the substantiation of the Health Claim on OO polyphenols. However, until now there has been no sufficient evidence supporting this effect, thus the studies analyzed were considered inconsistent. Moreover, in this trial, as well as in other trials discussed later [18,19,20,21,22,23,24], an important application of the chemical techniques is their use to test the dietary adherence, thus overcoming the self-reporting bias of food frequency questionnaires or food recalls to the study participants. 

Dose-dependent positive feedback in terms of LDL peroxidation reduction was also shown for phenolic compounds in a subpopulation of subjects from the EUROLIVE study [25].

Considering that oxidative stress also supports endothelial dysfunction, a post prandial consumption of HPC (400 mg/kg) and LPC (80 mg/kg) VOO was administered to hypercholesterolaemic volunteers [26]. Even if, also in this case, the phenolic composition of VOO was not sufficiently chemically characterized, the authors demonstrated the ability of high content of VOO phenolics to revert the impairment of the endothelial function during the post-prandial state. The mechanisms of action included the reduction of the oxidative stress measured as lipoperoxides (LPO) and 8-iso-F2α levels, and the increase of the nitric oxide (NO) metabolites, as demonstrated by biochemical analyses. However, LPO did not seem to be a reliable marker for lipid peroxidation, and the modulation of F2α-isoprostanes by phenolic content, different to what was found by Widmer et al. [27], but in accordance with other clinical trials [15,18,20], was not significant.

Apart from these clinical trials, Table 1 reports additional human studies correlating the chemical characterization of VOO phenolic compounds in terms of quantity and quality, with the antioxidant and anti-inflammatory (see below) abilities tested by chemical or biochemical and cellular assays. However, a list of additional clinical trials using OO polyphenols, non-restricted to the row form of olive oil and to the correlation with their chemical characterization, is reported by Flori et al. [28].

Concerning the EVOO phenolic antioxidant potential, total polyphenols quantification by the Folin-Ciocalteu and HPLC methods was correlated to the ability to protect against LDL oxidation; this effect correlated with the phenolic concentration of EVOO. Specifically, by an *ex vivo* study the authors demonstrated that, after incubation with plasma, EVOO phenols could be incorporated into LDL particles and exert their antioxidant activity [30]. The antioxidant ability of EVOO phenolics correlates to the prevention of cardiovascular diseases, as also reported in the PREDIMED study in which human subjects consumed EVOO or nuts in the context of an MD [38].

However, for the ability of VOO polyphenols to bind to LDL, some crucial aspects have to be taken into account when clinical trials were performed, e.g., the analysis of metabolites instead of EVOO primary species and the need to use a highly sensitive detection technique following an adequate extraction procedure in order to minimize the matrix effect. In fact, phenolic compounds undergo an intensive intestinal and hepatic metabolism after ingestion. For this reason, it could be better to consider metabolites instead of the primary species for the study of EVOO biological activity, even if their concentration is very low. Delatorre-Carbot et al. demonstrated that HPLC-ESI-MS/MS determination after solid phase extraction gave the best results in terms of T and HT metabolites quantification on LDL recovered from healthy subjects’ plasma after a post prandial administration of EVOO *Picual* variety of the O. europaea L. fruit [31]. Specifically, hydroxytyrosol monosulfate, hydroxytyrosol monoglucuronide, tyrosol sulfate, tyrosol glucuronide and homovanillic acid sulfate are able to bind to human LDL, reinforcing their antioxidant action *in vivo*. Another clinical trial investigating the effect of specific metabolites of OO phenols on human LDL lipid composition and peroxidation was conducted on a subsample population of the EUROLIVE study [32]. Specifically, VOO with HPC significantly increased the concentrations of hydroxytyrosol monosulfate and homovanillic acid sulfate (but not of tyrosol sulfate) in LDL particles and decreased the concentration of circulating markers of lipid peroxidation, including LDL particles (oxLDL), conjugated dienes and hydroxy fatty acids. Thus, an inverse relationship between the degree of LDL peroxidation and the concentrations of phenolic metabolites in LDL particles was observed.

Moreover, many other clinical trials studying the antioxidant potential of VOO phenolic compounds were performed, but only some of them reported a detailed chemical characterization. These trials were performed on healthy subjects and differed for their timing (sustained consumption vs. short-term/acute consumption). In the first case, the protective effect of VOO with a high polyphenols content was demonstrated on healthy volunteers who had undergone a sustained consumption of refined, common and VOO [19]. The ever-increased phenolic concentration in the three groups (refined < common < VOO), as reported by the Folin-Ciocalteau method and HPLC analysis, correlated with a more effective LDL protection from oxidation, as demonstrated by a decrease in oxidized LDL (oxLDL) and an increase of HDL cholesterol levels. In the short-term trial, healthy subjects consumed EVOO with HPC (486 mg/kg), MPC (133 mg/kg) and LPC (10 mg/kg) for four days [20]. As also recognized by the EFSA panel, a dose-dependent effect was observed between the EVOO polyphenols content with a decrease in LDL peroxidation and malondialdehyde (MDA) and an increase in the antioxidant glutathione peroxidase activity (GSH-Px) [16]. As indicated before, a not significant modulation was reported for 8-iso-prostaglandin F2α. Finally, an acute intake of HPC (366 mg/kg), MPC (164 mg/kg) and LPC (2.7 mg/kg) in healthy subjects demonstrated a direct correlation between the content of VOO phenolic compounds and a significant decrease in plasma oxLDL [16,18]. In this case, the 8-iso-prostaglandin F2α measurement did not show a dose-dependent effect relative to phenolic content variation too.

Regarding the studies in which an accurate chemical characterization of VOO polyphenols was related to their antioxidant ability tested not only by biochemical but also by cellular assays, different clinical trials were reported (Table 1). Specifically, a sustained consumption of VOO (161 mg/kg) vs. refined OO (14.7 mg/kg) was examined in stable coronary heart disease (CHD) patients with a diagnosis of hypertension [22]. In fact, the present study aimed to evaluate the impact of phenolic compounds not only on oxidative stress in stable CHD patients, but also on blood pressure in hypertensive and stable CHD patients. The authors demonstrated that VOO decreased oxLDL and increased GSH-Px, as compared to refined OO. This effect on the enzymes regulating the antioxidant status was also observed in elderly healthy subjects. The consumption of EVOO induced an enhancement of the endogen antioxidant system in healthy elderly people, in which an increased oxidative stress and an impaired antioxidant defense system were usually reported [29]. Moreover, the increased intake of phenolic compounds was also able to decrease the systolic blood pressure. However, only few human studies analyzed the positive effects of OO on blood pressure [39]; this could possibly be the reason why the EFSA could not draw a scientific substantiation for the relative claimed effect [16].

It is important to report also contrasting results obtained from a single-blind, randomized, crossover trial on Greek smokers undergoing a sustained consumption of two EVOO cultivars characterized by a HPC and a LPC. In fact, no differences were reported for total plasma resistance to oxidation, the ferric reducing ability of plasma, the concentrations of protein carbonyl, MDA, or lipid hydroperoxides [33]. This could be due to the relatively small size of the trial, and to its organization requiring a run-in period before the first treatment period and a washout between the two treatment periods. Due to a missed control for other fat sources than olives and oil-containing products, it was not possible to establish the effectivity in these periods. Finally, another clinical trial demonstrated the antioxidant ability of phenolic compound enriched EVOO to protect healthy postmenopausal women from oxidative DNA damage, as assessed by the comet assay in peripheral blood lymphocytes [23].

## 3. Correlation among EVOO Polyphenols Chemical Characterization, Anti-Inflammatory Potential and Biological Activities in Human Studies

The anti-inflammatory effect of VOO’s phenolic content was demonstrated in many pathological contexts such as CVD, chronic inflammatory disorders, such as the intestinal inflammation leading to colorectal cancer (CRC) development, and autoimmune diseases [2]. A clinical trial on stable CHD patients demonstrated the ability of VOO to reduce two pro-inflammatory markers, i.e., Interleukin 6 (IL-6) and C-Reactive Protein (CRP), as compared to refined olive oil (ROO), devoid of phenolic compounds [21]. 

Another study on women with mild hypertension, in which both the anti-inflammatory and antioxidant VOO properties were analyzed, demonstrated the ability of polyphenols enriched VOO to decrease the blood pressure and inflammatory markers, and improve the endothelial function, thus supporting a beneficial role for VOO polyphenols in CHD prevention [34]. 

Differences in the total polyphenols content, measured by the Folin-Ciocalteu method, was useful to understand the results of a human study based on VOO post prandial administration in patients affected by a metabolic syndrome. Specifically, the anti-inflammatory potential of VOO samples correlates with its polyphenols content. Thus, VOO with low polyphenol content was able to increase the postprandial release of endotoxin LPS (lipopolysaccharides) and its target genes like TLR4 (Toll Like Receptor 4) that, in turn, activates NF-κB signaling, promoting the secretion of pro-inflammatory cytokines and chemokines by PBMCs (peripheral blood mononuclear cells) like IL-1β and CXCL1 (chemokine C-X-C motif Ligand 1) at molecular level, as well as IL-6, whose significant up-modulation was found also in plasma samples [35]. 

Moreover, the high polyphenol content in EVOO, as measured by HPLC, is able to ameliorate the T2D patients’ metabolism. In fact, apart from the induction of an improvement in anthropometric measurements, EVOO enriched in phenolic content is also able to modulate some biochemical parameters, such as the fasting glucose and liver enzymes, along with inflammatory adipokines profile, in particular visfatin [24].

Furthermore, differences in phenolic content, measured by the Folin-Ciocalteux method, were also related to another important aspect of OO research, i.e., the maturation process in an *ex vivo* study on PBMCs from healthy subjects treated with *Koroneiki* cultivar (Greece). Specifically, the increase in phenolic compounds during maturation correlated with an increase in antioxidant and anti-inflammatory properties of the tested cultivar, as reported by results from DPPH, FRAP and copper-induced lipid oxidation tests, and by the decrement of some circulating pro-inflammatory cytokines (TNF-α and MCP-1) levels [36]. Moreover, the correlation of VOO phenolic content, identified by HPLC, and antioxidant activity, measured by ORAC assay, was investigated as related to the nutrigenomic effect in a clinical trial on pre/hypertensive patients, as discussed in a recent review [3]. Post prandial administration of VOO with HPC promoted several atheroprotective molecular mechanisms acting on genes related to cholesterol efflux in humans, as compared to VOO with an MPC. Among these genes, the increase in ABCA1 (one of the main transmembrane transporters for cholesterol efflux), PPAR-α (peroxisome proliferator-activated receptor-alpha) and PPAR-γ transcription factors controlling both lipid metabolism and inflammation, correlated with a decrease in oxidized LDL. Furthermore, ORAC values were directly correlated with the molecular induction of PPAR genes and their coactivator PPARBP (PPAR-binding protein). This could explain the increase in CD36 molecular expression found after HPC vs. LPC treatment in human white blood cells. In fact, CD36 is a scavenger receptor promoting the uptake of oxidized LDL. Finally, the enrichment of polyphenols in VOO induced, in these patients, the up-modulation of other transmembrane transporters for cholesterol efflux, i.e., SR-B1 [37]. 

Notwithstanding the reported studies analyzing the anti-inflammatory activity of VOO phenolic compounds (Table 1), a specific Health Claim was not released by the EFSA. In fact, even if a claimed effect on anti-inflammatory properties of olive biophenols exists in the context of diseases such as osteoarthritis or rheumatoid arthritis, the EFSA define it as general and not specific, also because it does not comply with the criteria laid down in Regulation (EC) No 1924/2006 [16].

An important consideration emerging from this paragraph is that studies correlating the chemical characterization and the anti-inflammatory properties of VOO phenolic compounds are still scanty. In fact, even if these techniques were used for other tested compounds from different plants [40,41,42,43,44,45], they did not find their application in VOO research until now. Thus, more research is needed to extend the use of chemical tests for the study of VOO anti-inflammatory properties. 

## 4. Conclusions

**Figure 1:**
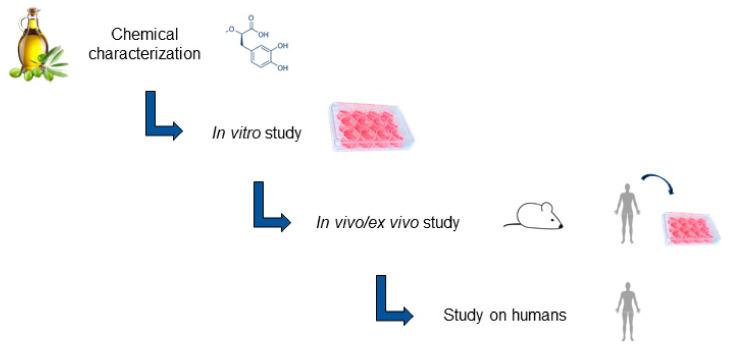
Systematic approach to study the EVOO beneficial activity on human health.

Considering the properties of EVOO as a functional food reported in this review, the process by which studies demonstrating VOO’s beneficial effects on human health can be translated into an approved Health Claim appears long, complex and requires a tight correlation between the chemical characterization of VOO’s beneficial compounds and their specific dose to be used in a defined physio-pathological context. A specific indication for the dosage is extremely important to avoid a shift toward pro-inflammatory and pro-oxidant activities typically associated with a high polyphenol intake [46]. Starting from the studies discussed in this review and summarized in Table 1, the urgent need of a systematic approach to study VOO’s beneficial properties comes up clearly. In fact, all the studies that analyzed VOO’s beneficial properties only by a unique approach (*in vitro* or *in vivo*, or *ex vivo* or human studies) and important aspects relative to the tested compounds (E/VOO vs. its by-products), the OO dose, OO maturation process, as well as geographical/climatic conditions, were often underestimated [47]. Moreover, many are the studies in which VOO is directly tested on humans, neglecting important data relative to the bioavailability. Additionally, some discrepancies for the reported results in human studies could be explained by different administration timings (short-term vs. sustained consumption). The application of different techniques also complicates the comparison of the results obtained from distinct studies. In fact, the use of chemical and/or biochemical/biological assays gives back specific scientific answers, and each assay is characterized by different features in terms of sensibility and specificity. For this reason, a correct choice of the assays to be used in each study is crucial. Thus, like the strict *iter* followed for drug discoveries and validation, a well-structured protocol to study functional foods is needed in which food compounds have to be chemically characterized first, and then sequentially tested by *in vitro*, *in vivo*/*ex vivo* assays, and finally on humans (Figure 1). 

Even if utopistic until now, this new systematic approach could be very useful to overcome one of the main drawbacks of the clinical trials, i.e., the lack of correlation with the results obtained in preliminary studies.

Finally, the need to extend the use of chemical assays to test the anti-inflammatory properties of VOO implies an important consideration. Once the results of these assays are validated by biological studies, one possible application could be the creation of specific devices able to simply discriminate functional foods from foods without beneficial effects on human health. These devices could have the typical advantages of chemical assays, i.e., saving time and costs, and their use could be especially important for OO producers, helping them to better manage OO production, with a good economic relapse on the market.

## Figures and Tables

**Table 1:** Summary of human studies analyzing the antioxidant and anti-inflammatory properties of EVOO/VOO by a different combination of chemical, biochemical and biological assays, starting from the chemical characterization of the OO phenolic profile. 8-iso-PGF2α: 8-epi-isoprostane prostaglandin F2α; ABTS: 2,2’-Azinobis-(3-ethylbenzothiazoline-6-sulfonic acid); ALT: alanine transaminase; AST: aspartate transaminase; CAT: catalase; CRP: C-reactive protein; DPPH: 2,2-diphenyl-1-picrylhydrazyl; ELISA: enzyme-linked immunosorbent assay; EVOO: extra virgin olive oil; FRAP: ferric reducing antioxidant power; GR: GSH reductase activity; GSH: glutathione; GSH-px: GSH peroxidase; H_2_O_2_: hydrogen peroxide; HDL-C: high density lipoprotein cholesterol; HPC: high polyphenols content; HT: hydroxytyrosol; LDL-C: low density lipoprotein cholesterol; LPC: low polyphenols content; LPO: levels of lipoperoxides; MDA: malondialdehyde; MHT: 3-O-methylhydroxytyrosol; MPC: moderate polyphenols content; NO(x): nitrates/nitrites; OLAB: oxLDL serum antibodies; oxLDL: oxidized LDL; ROO: refined olive oil; SOD: superoxide dismutase; T: tyrosol; T2D: type 2 diabetes mellitus; TAC: total antioxidant capacity; TAG: triglycerides; TAS: total antioxidant status; TBARS: thiobarbituric acid reactive substances assay; VOO: virgin olive oil.

Sample/ Treatment	Human Studies		Antioxidant Test		Anti-Inflammatory Test	Other Biological Analysis	REF
	*Ex Vivo* Study	Clinical Trial	Chemical Assays	Biochemical/ Cellular Assays	Biochemical/ Biological Assays		
Sustained consumption of HPC/MPC/LPC VOO on healthy subjects following a randomized, crossover, controlled trial		Plasma, serum and urine	Total F2α-isoprostanes, C18 hydroxy fatty acids, T and HT	Oxidative damage, endogenous and exogenous antioxidants		Glucose, total cholesterol, HDL-C, LDL-C and TAG	[15]
Sustained consumption of EVOO vs. ROO in healthy subjects following a randomized, crossover trial				OxLDL		Glucose, total cholesterol, HDL-C, LDL-C, TAG and extensive study on HDL	[25]
Post prandial consumption of HPC vs. LPC VOO in hypercholesterolemic subjects		Plasma and serum		8-epi-F_2α_, LPO and NO(x)		Ischemic Reactive Hyperemia and lipid parameters	[26]
Sustained consumption of EVOO in healthy elderly subjects following a randomized trial		Plasma and erythrocyte	TAC, OH-Tyr	CAT, SOD, GSH-px		Glucose, total cholesterol, HDL-C, LDL-C and TAG	[29]
EVOO vs. ROO	Plasma from healthy subjects			Copper sulfate-oxidized LDL/TBARS			[30]
Post prandial administration of EVOO produced from the *Picual* variety of *O. europea* L. fruit in healthy subjects		Plasma		Analysis of T and HT metabolites ability to bind the human LDL			[31]
Sustained consumption of HPC/MPC/LPC VOO on healthy subjects following a randomized, crossover, controlled trial		Plasma, serum and urine	C18 hydroxy fatty acids and LDL cholesterol-uninduced conjugated dienes	Analysis of LD oxidation		Total Cholesterol, HDL-C and LDL-C	[32]
Sustained consumption of refined, common and virgin olive oil in healthy subjects following a placebo-controlled, double blind, cross-over, randomized, trial		Plasma, serum and urine	HT and T	Analysis of LD oxidation, LDL resistance to oxidation		Glucose, total Cholesterol, HDL-C and LDL-C	[19]
Short-term consumption of HPC/MPC/LPC EVOO on healthy subjects following a double-blind, randomized, crossover trial		Plasma, serum and urine	8-oxo-dG, MDA, 8-iso-PGF2α, T, HT and MHT	OxLDL, GR and GSH-Px		Total Cholesterol, HDL-C and LDL-C	[20]
Sustained consumption of HPC/MPC/LPC VOO on healthy subjects following a randomized, crossover, controlled study		Plasma, serum and urine	T, HT and MHT	oxLDL and 8-iso-PGF2α		Glucose, total Cholesterol, HDL-C and LDL-C	[18]
Sustained consumption of EVOO and ROO on patients with stable CHD following a placebo controlled, crossover, randomized trial		Plasma, serum and urine	ABTS, T, HT and MHT	OxLDL, OLAB, TBARS, TAS and GSH-Px		Glucose, total cholesterol, HDL-C, LDL-C, TAG and blood pressure	[22]
Sustained consumption of *Koroneiki* or *Tsounati* cultivar on healthy subjects following a single-blind, randomized, crossover trial		Plasma and serum	FRAP	TBARS, lipid hydroperoxides, total plasma resistance to oxidation and protein carbonyls			[33]
Short-term consumption of two Italian EVOO blends with high or low phenolic content on postmenopausal women following a randomized, cross-over trial		Plasma and urine	ABTS, HT and homovanillyl alcohol	Analysis of DNA breaks and oxidized bases by comet assay, lymphocytes exposure to H_2_O_2_		Plasma biomarkers analysis	[23]
Sustained consumption of EVOO vs. ROO on stable CHD patients following a placebo-controlled, crossover, double-blind, randomized trial		Plasma, serum and urine	T, HT and MHT		Analysis of inflammatory markers by ELISA and immunoturbidimetry	Glucose, total cholesterol, HDL-C, LDL-C, TAG	[21]
Sustained consumption of EVOO vs. ROO on women with mild hypertension following a double-blind, randomized, crossover study		Plasma and serum		OxLDL, NO(x) and other markers for endothelial function	CRP analysis by enzyme immunoassay	Systolic and diastolic blood pressure	[34]
Post prandial administration of VOO with high, intermediate and/or low phenol content in metabolic syndrome patients following a randomized crossover study		Plasma, serum and PBMCs			Analysis of pro-inflammatory markers at molecular and protein level	Lipid analysis and biochemical determinations	[35]
Short-term consumption of EVOO vs. VOO on overweight and non-insulin treated T2D patients		Plasma, serum and PBMCs	HT		Analysis of pro-inflammatory cytokines and adipokines by ELISA and immunoassay kit	Glucose, total cholesterol, HDL-C, LDL-C, TAG, ALT and AST	[24]
*Koroneiki* cultivar from Greece		PBMCs from healthy subjects	Folin-Ciocalteu	DPPH and FRAP	Antioxidant activity of extracts in copper induced serum lipid oxidation	Analysis of pro-inflammatory cytokines by ELISA	[36]
Post prandial consumption of MPC vs. HPC VOO in pre/hypertensive patients following randomized, double-blind, crossover, controlled trial		Plasma, serum and WBCs	ORAC and FRAP		Expression analysis of genes involved in the cholesterol efflux	Total cholesterol, HDL-C, LDL-C, TAG	[37]
